# Importance of environmental signals for cardiac morphological development in Atlantic salmon

**DOI:** 10.1242/jeb.247557

**Published:** 2024-10-18

**Authors:** Marco A. Vindas, Vilde Arntzen Engdal, Simona Kavaliauskiene, Ole Folkedal, Erik Höglund, Marta Moyano, Øyvind Øverli, Michael Frisk, Ida B. Johansen

**Affiliations:** ^1^ Department of Preclinical Sciences and Pathology, Faculty of Veterinary Medicine, Norwegian University of Life Sciences, 1433 Ås, Norway; ^2^ Institute for Experimental Medical Research, University of Oslo and Oslo University Hospital Ullevål, 0450 Oslo, Norway; ^3^ K.G. Jebsen Center for Cardiac Research, University of Oslo, 0450 Oslo, Norway; ^4^ Research Group of Animal Welfare, Institute of Marine Research, 5984 Matredal, Norway; ^5^ Niva, Norwegian Institute for Water Research, 0579 Oslo, Norway; ^6^ Center of Coastal Research, University of Agder, 4604 Kristiansand, Norway; ^7^ Department of Animal and Aquacultural Sciences, Faculty of Biosciences, Norwegian University of Life Sciences, 1433 Ås, Norway

**Keywords:** Cardiac remodeling, Smoltification, Diastolic dysfunction

## Abstract

The hearts of salmonids display remarkable plasticity, adapting to various environmental factors that influence cardiac function and demand. For instance, in response to cold temperature, the salmonid heart undergoes growth and remodeling to counterbalance the reduced contractile function associated with dropping temperatures. Alongside heart size, the distinct pyramidal shape of the wild salmonid heart is essential for optimal cardiac performance, yet the environmental drivers behind this optimal cardiac morphology remain to be fully understood. Intriguingly, farmed salmonids often have rounded, asymmetrical ventricles and misaligned bulbi from an early age. These deformities are noteworthy given that farmed salmon are often not exposed to natural cues, such as a gradual temperature increase and changing day lengths, during critical developmental stages. In this study, we investigated whether natural environmental conditions during early life stages are pivotal for proper cardiac morphology. Atlantic salmon were raised under simulated natural conditions (low temperature with a natural photoperiod; SimNat) and compared with those reared under simulated farming conditions (SimFarm). Our findings reveal that the ventricle shape and bulbus alignment in SimNat fish closely resemble those of wild salmon, while functional analyses indicate significant differences between SimNat and SimFarm hearts, suggesting diastolic dysfunction and higher cardiac workload in SimFarm hearts. These findings highlight the profound influence of environmental factors such as water temperature and photoperiod on the structural development of the salmonid heart, underscoring the importance of early environmental conditions for cardiac health.

## Teaser

**Summary:** Early-life exposure to natural conditions optimizes the pyramidal shape and bulbus alignment of Atlantic salmon hearts, highlighting the importance of water temperature and photoperiod for heart morphology.

## INTRODUCTION

Animals that undergo dramatic habitat shifts during their life cycle are characterized by complex adaptations which help in their survival. This includes physiological adaptations of vital organ systems that are synchronized by environmental signals, such as day length and temperature (McCormick, 2012; West and Wood, 2018). Anadromous salmonids such as Atlantic salmon (*Salmo salar*) have complex life cycles, with spawning and juvenile stages taking place in fresh water, before migration to sea water to grow and mature (Stefansson et al., 2008). Furthermore, they tend to live in cold temperate areas where they remain active at a range of temperatures (Jonsson and Jonsson, 2009). Thus, anadromous salmonids need to adjust to variation in both salinity and temperature, and have evolved complex physiological adaptations allowing such a lifestyle (Eliason and Anttila, 2017; McCormick, 2012). For example, in rainbow trout (*Oncorhynchus mykiss*), the heart grows and remodels in response to cold temperatures to compensate for the negative effect of decreasing temperature on contractile function (Graham and Farrell, 1989; Keen et al., 2016, 2017; Klaiman et al., 2011). Similarly, in the same species, the transition from fresh water to sea water is associated with myocardial growth (compactum), which generates increased stroke volume and cardiac output (Brijs et al., 2017). This cardiovascular response is believed to compensate for hemodynamic changes associated with increased salinity, as well as an increased demand for circulation of osmoregulatory organs (Brijs et al., 2015). In this context, there are indications that cardiac preparations for the sea water transition may be initiated when the fish go through their transformation for seawater adaptation (i.e. smoltification). In wild Atlantic salmon, smoltification is triggered by photoperiod and temperature cues (i.e. a gradual increase in day length and water temperature) and involves a range of behavioral, physiological and morphological changes preparing the fish for downstream migration and sea water entry (Stefansson et al., 2008). For example, relative heart size increases from parr to smolt stage, even before the salmon transition to sea water (Graham and Farrell, 1992; Leaonard and McCormick, 2001; McCormick, 2012).

While heart size plays a crucial role, heart shape is equally pivotal for optimizing cardiac and physical performance (Claireaux et al., 2005). This is evident in certain athletic fish species, such as salmonids and tunas, that exhibit a distinct heart morphology characterized by an elongated pyramidally shaped ventricle (Anttila and Farrell, 2011). Unlike hypertrophic remodeling, the factors driving morphological alterations and development of this geometrically advantageous heart shape remain largely unexplored.

Interestingly, from early stages of their life cycle, farmed salmon exhibit a substantially different heart shape from that of their wild counterparts (Engdal et al., 2024). In general, their ventricles are more rounded and asymmetrical, bulbi are misaligned, and a notable accumulation of fat deposits on both bulbus and ventricle is often apparent (Brijs et al., 2020; Frisk et al., 2020; Poppe et al., 2003). There are several possible explanations for the differences in heart shape between wild and farmed salmon. For example, farmed salmon have undergone intense selective breeding for decades, leading to significant genetic differences from their wild counterparts. Additionally, farmed salmon have a markedly different diet, lower levels of physical activity and are often deprived of natural environmental cues and reared under conditions that diverge significantly from the natural environment experienced by their wild counterparts. Commercial Atlantic salmon hatcheries, for instance, typically rear salmon under continuous light and at elevated temperatures to promote accelerated growth during the freshwater stage (Handeland et al., 2003; Steffanson et al., 1991). Notably, two recent field studies performed on fish nearing harvest size, after rearing at commercial sea farms, indicate that cardiac morphological deviations in farmed salmonids can be linked to elevated temperatures during early rearing (Brijs et al., 2020; Frisk et al., 2020). For example, Brijs et al. (2020) showed that the severity of morphological deviations of the hearts of farmed rainbow trout could be linked to hatchery of origin and these deviations were more prevalent in fish from hatcheries using elevated rearing temperatures (10–15°C). Similarly, Frisk et al. (2020) linked elevated temperature in the hatchery to more severe ventricular morphological deviations in commercial Atlantic salmon nearing harvest. Thus, these snapshot analyses from the field suggest that rearing strategies (i.e. the use of warm water) contribute to development of deviating heart shapes. They specifically point to environmental factors from hatching to smoltification as potential key influencers of cardiac remodeling and development. However, these field studies are observational and lack controlled conditions, making it difficult to exclude the possibility that observed effects are due to genetic differences or other variables inherent to commercial fish populations. Thus, there is a need for controlled experimental studies to rigorously test the influence of environmental factors on the geometrical remodeling of the heart in salmon. Of note, environmental factors regulating physiological development tend to interact. For example, whereas a change in photoperiod is a key environmental trigger of most physiological aspects of smolt development in nature (Hoar, 1988), the ability of photoperiod to affect smoltification is likely to be dependent on temperature, and temperature has been proposed as a controlling factor in the smoltification process (Fry, 1971; McCormick et al., 2000). In this context, we believe that photoperiod and temperature may interact in the regulation of cardiac development.

Here, we tested the hypothesis that exposure to a more natural environmental regime during early rearing stages is necessary for proper morphological development of the heart in Atlantic salmon. We reared Atlantic salmon under either simulated natural conditions (i.e. low temperature and a simulated natural photoperiod; SimNat) or simulated farmed conditions (i.e. elevated temperature and periods of continuous photoperiod; SimFarm) and compared their heart morphology throughout the experimental period. Heart morphology was compared with that of wild Atlantic salmon fry, parr and smolt. Overall, SimNat fish exhibited a closer resemblance to wild salmon than SimFarm hearts, clearly indicating that environmental signals, such as water temperature and photoperiod, play significant roles in the geometric modelling of the salmonid heart. Importantly, functional analyses uncovered significant differences between SimFarm and SimNat hearts, suggesting the adverse effects of altered morphology on cardiac health in SimFarm fish.

## MATERIALS AND METHODS

### Ethics statement

The experiments were performed in accordance with current Norwegian law for experimentation and procedures on live animals and were approved by the Norwegian Food Safety Authority under permit number 24848.

### Fish husbandry

**Fig. 1.**
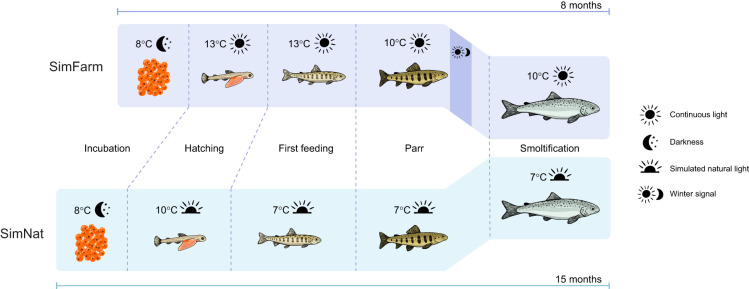
Schematic representation of the experimental design time line for the Atlantic salmon groups reared in simulated natural (SimNat) and simulated farmed (SimFarm) conditions.

The fish used in this experiment were from the AquaGen Atlantic QTL-innOva SHIELD strain. A total of 3600 fish were hatched in two batches (*n*=1800 per treatment) on site at the Matre Research Station facilities, Institute of Marine Research, Norway. The aim of the experiment was to compare how rearing conditions affect heart morphology by coordinating a common smoltification for fish reared under simulated natural (SimNat) and simulated farmed (SimFarm) conditions. As simulated natural conditions are associated with slower growth than the simulated farmed conditions, the SimNat group batch was hatched in December 2019, while the SimFarm group batch was hatched 10 months later (September 2020), which resulted in both groups smoltifying in May 2021. During egg incubation, the conditions were the same for the two groups (8°C and total darkness), but from hatching to the start of feeding, the SimNat group was kept at 10°C and the SimFarm group at 13°C, both at 24 h light. After the start of feeding, the SimNat group was kept at 7°C and on a natural photoperiod until smoltification in May 2021. Meanwhile, the SimFarm group was kept at 13°C and with 24 h light for 6 months, followed by a 12 h:12 h light:dark period (winter signal for 6 weeks) before returning to a 24 h light regime until smoltification in May 2021. At the onset of the winter signal, temperature was also decreased to 10°C for the SimFarm fish to avoid extreme size disparities between the groups. Even though it took more time for the SimNat fish to grow, compared with the SimFarm fish, temperature differences resulted in the two groups being very close in size. Both treatment groups were kept on a continuous flow of filtered, UV-C treated and aerated water, which ensured oxygen saturation >85% levels at all times and prevented waste products from accumulating. Triplicates for each treatment were maintained until smoltification. Circular 100 l tanks were used until the fish reached 1.54 g, at which point they were transferred to square 500 l tanks until smoltification. The fish were fed to satiation daily with commercial pellets (Nutra Olympic, Skretting, following the manufacturer's growth tables), between 14:00 h and 18:00 h via automatic feeding devices. All fish were vaccinated after reaching approximately 30 g with AQUAVAC^®^ PD7 (MSD Animal Health) following standard salmon aquaculture procedures. See Fig. 1 for a schematic illustration of the experimental design.

### Sampling

The treatment groups were sampled at four time points throughout the experimental period: (1) at the fry stage, (2) during the parr stage (both experimental groups were sampled at the onset of the winter signal for the SimFarm group), (3) at the estimated start of smoltification, and (4) at the end of the smoltification (fish for this sampling were sampled on three different occasions during 1 week, but the data were pooled as they all represent the same time point). On all sampling occasions, four fish were randomly chosen from each tank for a total of 12 fish per time point/treatment, except for the first time point (the fry stage point) at which 15 fish were sampled (five per tank). At all samplings, fish were netted from their home tanks and immediately euthanized with a lethal dose of buffered MS-222 (Sigma-Aldrich) at a concentration of 2 g l^−1^ until completely unresponsive and motionless (within approximately 30 s). Fish were rapidly weighed, fork length was measured, and a blood sample was taken from the caudal vein using a 23G, 1 ml syringe containing the anticoagulant EDTA. For hematocrit (Hct) measurements, blood was collected in heparinized capillary tubes (Fisher Scientific, Waltham, MA, USA) that were sealed and centrifuged in a Hct centrifuge for 5 min at 3000 rcf. The proportion of red blood cell volume to whole-blood volume was measured twice per sample. The rest of the blood was centrifuged for 10 min at 9289 rcf at 4°C, before plasma samples were frozen and stored at −80°C for later cortisol analysis. The heart was then removed, rinsed in PBS and transferred to 20 mmol l^−1^ KCl solution. Once the heart ceased beating, it was transferred to buffered 10% formalin solution and stored at 4°C.

### Wild fish sampling

Atlantic salmon fry and parr were captured by electrofishing at Hagnesvassdraget, a western tributary of Numedalslågen in Kvelde, Norway (59.19753°N, 10.10184°E) in August 2021. A total of 24 fry (mass: 2.92±1.32 g, length: 6.57±0.71 cm, means±s.d.) and 28 parr (mass: 12.53±5.79 g, length: 10.75±3.1 cm) were euthanized and sampled as explained above.

Downstream migrating wild Atlantic salmon smolts were caught at two points in the River Nidelva, Agder, Norway (58.41540°N, 8.74242°E) in May 2021. First, smolts were collected using a modified fyke net placed in the Songeelva river, a tributary of the Nidelva river, 25 km upstream of the river mouth. Second, fish were caught in a Wolf trap located at the entrance of the surface fish passage at Rygene hydropower plant, 9.4 km downstream of the river mouth. In both cases, fish were collected from the trap and transported to collection tanks at the Rygene hydropower plant. A total of 44 smolt (mass: 34±6.87 g, length: 21.44±1.31 cm) were euthanized and sampled as explained above.

### Plasma cortisol

Plasma cortisol samples were analyzed using a commercially available DetectX^®^ cortisol enzyme immunoassay kit (Arbor Assays, Ann Arbor, MI, USA) following the manufacturer’s protocol. The absorbance of the prepared ELISA plate was read in a plate reader at 450 nm and the concentrations were calculated using a four-parameter logistics curve.

### Imaging and analysis of cardiac morphology

Fixated hearts were photographed from the left lateral and ventrodorsal projections inside a Styrofoam box (internal H×W×L 24×21×27 cm) lit by an internal LED light (Northlight LEDlamp, art. no. 36-6465). Photographs were captured with a Cannon EOS 4000D camera with an EF-S 18-55 III lens mounted on the Styrofoam box. Thus, all hearts were photographed from the same angles and under standardized light conditions. All heart measurements were calculated and analyzed according to Engdal et al. (2024). Specifically, the ventricular height:width ratio was analyzed from the ventrodorsal projection by dividing the ventricle length (from apex to bulbus) by the ventricle width (widest part of ventricle). In the left lateral projection, ventricular symmetry was quantified by measuring the angle between the ventricular vertical axis and the axis running from the ventriculobulbar groove to the left dorsal ventricular apex. In the same projection, alignment of the bulbus arteriosus was quantified by measuring the angle between the bulbular horizontal axis and the ventricular vertical axis. Finally, the bulbus width:ventricular width ratio was quantified from the ventrodorsal projection by dividing the bulbus width (widest part of the bulbus) by the ventricle width (widest part of the ventricle). All photos were analyzed using Fiji (Schindelin et al., 2012).

### Magnetic resonance imaging (MRI) of fixed ventricles

Fixed hearts were placed in Dulbecco's phosphate-buffered saline (DPBS) with 0.5 mol l^−1^ Magnevist^®^ (Bayer Schering Pharma, Berlin, Germany) for 1–2 days. Then, the hearts were dried with paper tissue and filled with Fomblin^®^ Y LVAC 06/6 (Solvay Specialty Polymers Italy SpA, Bollate, Italy) through the atrium prior to mounting in 15 ml plastic tubes using cotton soaked in Fomblin^®^. MRI scans were performed on a 9.4 T MRI system equipped with Avance Neo console and Paravision 360 software (Bruker Biospin, Ettlingen, Germany) and a 19 mm quadrature-driven birdcage RF coil (Rapid Biomedical, Rimpar, Germany). A 3D gradient echo (FLASH) sequence was used for acquisition with an *XYZ* resolution of 25×25×200 μm, TR/TE of 35/6 ms, flip angle of 30 deg and averaging of 12.

### MRI image analysis

For compactum thickness analysis, five ventricular sections were selected: the section at the widest part of the ventricle, and four additional sections toward the apex with 5% increment of the total ventricular length. This resulted in five images within 20% of the total length of the ventricle (Fig. S1I). Image analyses were performed using Fiji. The total ventricular area was semi-automatically selected using the ‘magic wand’ function. Any irregularities due to the presence of air bubbles or tearing of the tissue were manually removed prior to further analysis. The spongiosum was manually selected, and the compactum was then selected as the difference between the whole ventricle and the spongiosum. Compactum thickness was measured using the ‘local thickness’ function in Fiji. The resulting thickness map was inspected manually for any irregularities, and mean thickness of the compactum was measured as an average of all pixels in the thickness map (Fig. S1II). To adjust for differences in ventricle size between hearts, compactum thickness was normalized to the square root of the spongious area in each section. The normalization is based on the approximation of the spherical geometry of the heart, and the assumptions that pressure and wall stress do not depend on heart size (Norton, 2001).

Volume analysis of different heart compartments was performed using a 3D Slicer 5.2.1 software (Fedorov et al., 2012). Whole-heart segmentation was performed using automatic thresholding (Otsu method) followed by manual inspection for air bubbles and selection of the lumen. Both the bulbus and atrium were manually removed from the selection to obtain ventricular measurements only. The ventricular lumen segment was defined as the difference between filled and empty (after automatic thresholding) ventricular segments. Segment statistics were automatically obtained using a built-in function in the 3D Slicer.

### Echocardiography

The functional effects of altered heart morphology were examined in a subset of SimFarm (*n*=10) and SimNat (*n*=10) individuals by echocardiography (Fig. S2). These individuals are part of a subgroup of fish that were kept in their home tanks under the same described conditions, except for water salinity. That is, the tanks were switched to seawater (33 ppt) 48 h before echocardiography measurements. Fish were anesthetized one by one, in 150 mg l^−1^ MS-222. When locomotory and gill movements ceased, individuals were transferred to a V-board where gills were irrigated with temperature-controlled water (10°C) containing 75 mg l^−1^ MS-222 to maintain light sedation. Echocardiography was performed as described by Becker et al. (2024) with a linear probe (GE 12L-RS at 13 MHz) connected to a Vivid iq ultrasound system (GE Vingmed Ultrasound A/S, Horten, Norway). The probe was placed along the fish's longitudinal axis using the atrio-ventricular (AV) valve, ventricular dorsal apex and ventro-bulbar (VB) valve of the heart as reference points for standardize imaging projections. Cardiac structure was visualized with b-mode, and hemodynamics (AV and VB blood flow velocities) were recorded in pulsed-wave Doppler mode. Every single recording lasted for at least three heartbeats and the whole imaging protocol took less than 10 min per fish. Images were analyzed with EchoPAC v.203 (GE Vingmed) and all parameters presented are an average of three heartbeats from each individual. Diastolic atrial and ventricular areas were obtained from b-mode recordings. Information about systolic function was obtained by assessing outflow tract diameter (b-mode) and outflow tract blood flow velocities with pulsed wave (PW) Doppler. These measurements were used to calculate the outflow tract pressure gradients and blood velocity time integral to allow estimation of cardiac output as described by Becker et al. (2024) and Ma et al. (2019). Early and late diastolic AV valve velocities (E- and A-wave, respectively) as well as their ratio (E:A) were measured during ventricular filling to obtain information about diastolic function. Isovolumetric relaxation time was analyzed to obtain additional information about diastolic function. Analyses of all echocardiography parameters, except cardiac output and stroke volume, were performed using built-in algorithms in EchoPAC (GE Vingmed).

### Statistical analysis

RStudio software 4.0.4 (R Development Core Team, https://www.r-project.org) was used for the statistical analyses. The statistical packages ‘nlme’ and ‘MuMIn’ were used for exploratory linear mixed effect models (LME). The heart morphology measurements (including volume and thickness), cortisol, Hct and biometric data were analyzed using a LME, with treatment (wild versus SimNat versus SimFarm) and time (fry stage, parr and end of the smoltification period) as categorical independent variables or treatment (SimNat versus SimFarm) and time (fry stage, parr stage, start of the smoltification and end of the smoltification) as categorical independent variables with both LMEs including tank as a random effect for the experimental groups. In addition, the surface area and volume MRI measurements at smoltification were analyzed using a LME, with treatment (wild versus SimNat versus SimFarm) as a categorical independent variable and fish as a random effect. The initial LME models allowed the independent variables to interact. However, the final model was selected based on the lowest Akaike information criterion (AICc) score, i.e. the model with the best data fit when weighted against model complexity. Visual inspection of the qqnorm and residual plots to check the assumptions of normality and homoscedasticity confirmed that these models conformed to these assumptions. Interactive effects between treatment and test were assessed using a Tukey–Kramer honestly significant differences *post hoc* test. Functional echocardiography data were analyzed with a Student's *t*-test between SimFarm and SimNat. A correlation matrix using the R package HMSC with Benjamini–Hochberg *P*-value correction was used to explore interactions between cardio somatic index (CSI), morphological measurements and functional echocardiography data. Significance was assessed as *P*≤0.05.

Raw data are given in Dataset 1.

## RESULTS

### Biometric differences between SimFarm, SimNat and wild salmon

Both treatment (body mass: χ^2^_1_=36.6, *P*<0.001 and length: χ^2^_1_=131, *P*<0.001) and time (body mass: χ^2^_2_=1283, *P*<0.001 and length: χ^2^_2_=2089, *P*<0.001) and their interaction (body mass: χ^2^_2_=7.53, *P*<0.001 and length: χ^2^_2_=71.9, *P*<0.001), influenced body mass and body length throughout the experiment. Whereas both groups grew heavier and longer with time, the SimFarm fish were consistently heavier and longer than the SimNat fish throughout the experimental period (Table 1).

**Table 1.**
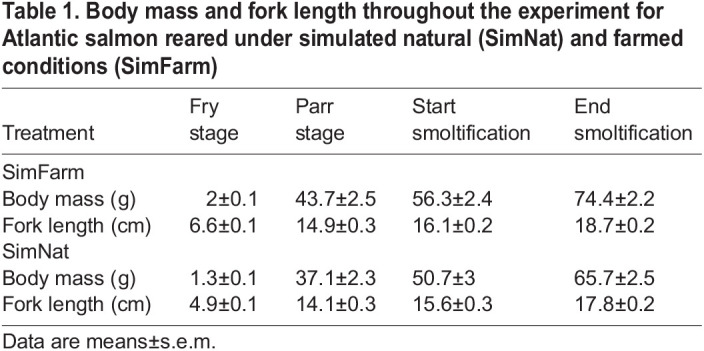
Body mass and fork length throughout the experiment for Atlantic salmon reared under simulated natural (SimNat) and farmed conditions (SimFarm)

At the end of the smoltification period, we found a significant difference in the relative ventricular mass (RVM=ventricle mass/body mass×100; χ^2^_2_=159, *P*<0.001) between all groups. Specifically, wild fish had a significantly lower RVM (0.16±0.006) compared with both SimNat (0.08±0.003) and SimFarm fish (0.09±0.003; *P*<0.001, for both). There were no differences in RVM between SimNat and SimFarm fish. Similarly, there was a significant effect of treatment on the CSI (χ^2^_2_=140, *P*<0.001) at the end of the smoltification period. That is, CSI was higher in wild fish (0.224±0.005) compared with both SimNat (0.146±0.003) and SimFarm fish (0.169±0.003; *P*<0.001, for both), with SimNat having lower CSI (*P*<0.001) than SimFarm fish.

### Plasma cortisol

While there was no treatment effect (χ^2^_1_=1.74, *P*=0.19) on plasma cortisol levels, there were significant time (χ^2^_2_=8.95, *P*<0.001) and interaction (χ^2^_2_=9.94, *P*=0.007) effects. Specifically, the plasma cortisol levels were higher in SimNat fish at the start of the smoltification period than in SimFarm fish (Fig. S3).

### Hematocrit

There were no significant differences (χ^2^_1_=0.002, *P*=0.96) in Hct values between SimFarm (52.8±1.8) and SimNat (52.7±1.7) fish.

### Fish reared in SimNat conditions develop a heart morphology that resembles that of wild salmon

**Fig. 2.**
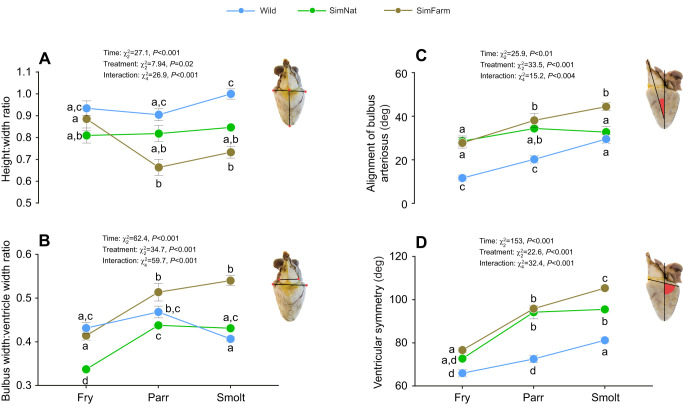
**Heart morphology measurements throughout the freshwater life stages (fry, parr and end of smoltification) for Atlantic salmon reared under SimFarm and SimNat conditions or captured from the wild.** (A) Height:width ratio, (B) bulbus width:ventricle width ratio, (C) alignment of bulbous arteriosus and (D) ventricular symmetry (means±s.e.m.). Linear mixed effects (LME) model statistics are given in each panel and lowercase letters represent Tukey *post hoc* differences between groups at all time points.

Heart morphology differed dramatically between the groups at smoltification, but some of these differences were apparent during the fry and parr stages. Firstly, while ventricular height:width ratio was not different between groups at the fry stage, it decreased in SimFarm compared with wild fish (*P*=0.007) during the parr stage. Furthermore, the height:width ratio was higher in wild than in both SimNat (*P*=0.001) and SimFarm (*P*<0.001) smolts, indicating that wild fish develop more elongated and narrow ventricles compared with experimental fish, and that rounding of the ventricle develops during the parr stage in SimFarm fish. Of note, whereas the height:width ratio remained stable throughout all sampling points in SimNat and wild fish, it decreased in SimFarm fish between the fry and the parr stages (*P*=0.001) as well as between the fry and smolt stages (*P*<0.001). Thus, SimFarm ventricles became gradually more rounded throughout the freshwater phase (Fig. 2A).

Bulbus:ventricle width ratio – a measurement of relative bulbus size – increased in both SimFarm (*P*<0.001) and SimNat (*P*<0.001) fish from fry to smolt and was consistently higher in SimFarm than in SimNat fish throughout the freshwater phase (*P*_fry_=0.001, *P*_parr_=0.02 and *P*_smolt_<0.001). Bulbus:ventricle width ratio appeared more stable in wild fish and was higher than in SimNat fish at the fry stage (*P*<0.001) and lower than in SimFarm fish at smoltification (*P*<0.001; Fig. 2B).

Alignment of the bulbus arteriosus angle was consistently higher in SimFarm compared with wild fish (*P*<0.001 for all time points), but both groups experienced a gradual increase in this angle throughout the period, indicating that the bulbus becomes less aligned with time (Fig. 2C). In SimNat fish, this angle was larger than in wild fish at the fry stage (*P*<0.001) but did not increase further throughout the freshwater phase. Thus, at smoltification, this angle was lower in SimNat than in SimFarm fish (*P*=0.002), but there was no difference between SimNat and wild fish (*P*=0.94).

Finally, the ventricular symmetry angle increased from fry to smolt stage in all groups but the ventricles of SimFarm fish were consistently more asymmetric than those of wild fish (i.e. larger ventricular symmetry angle; *P*<0.001 for all time points). Ventricular symmetry was similar in SimNat and SimFarm fish until the smolt stage, where ventricular asymmetry was more pronounced in SimFarm than in SimNat fish (*P*=0.005). Furthermore, ventricular symmetry angle was also higher in SimNat than in wild fish at both the parr (*P*<0.001) and smolt stages (*P*<0.001; Fig. 2D).

All in all, ventricles of SimNat fish tended to be more similar to those of wild fish compared with SimFarm fish.

### Differences between SimNat and SimFarm fish are already present at the start of the smoltification period

In order to increase our understanding of the temporal resolution of morphological development during the freshwater stage, we included a sampling point for the experimental groups during the start of the smoltification period. While differences in ventricle roundness (i.e. height:width ratio) were only evident at the end of the smoltification period, all other measurements were already significantly different (bulbus:ventricle width: *P<*0.001, alignment of the bulbus arteriosus angle: *P=*0.04 and ventricular symmetry angle: *P<*0.001) between SimNat and SimFarm fish at the start of the smoltification period (Fig. S4).

### Tissue composition and lumen properties of the ventricles

**Fig. 3.**
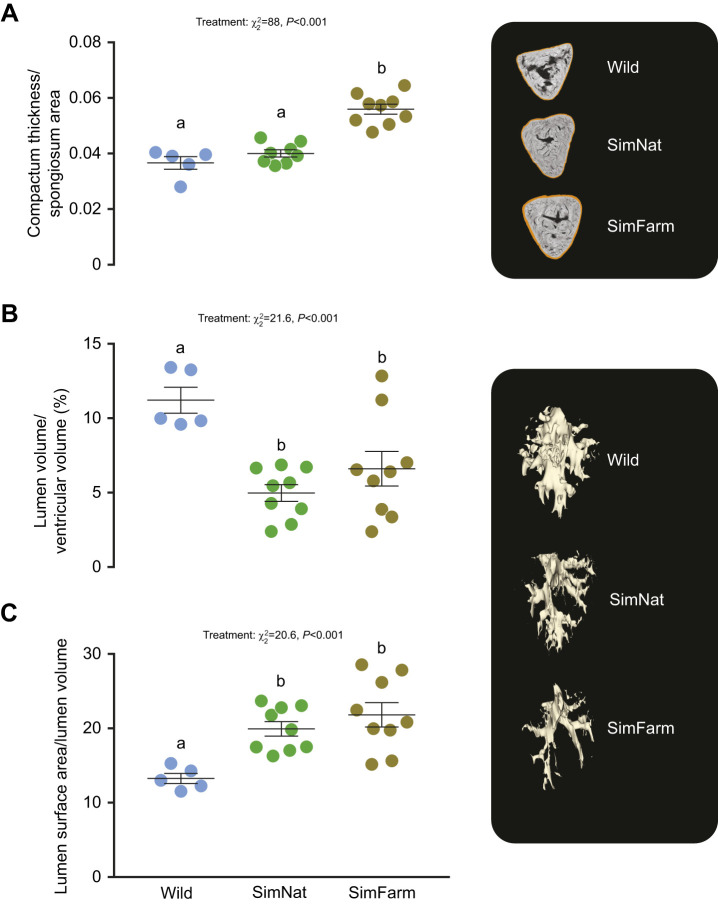
**Ventricle measurements taken at the end of the smoltification period in Atlantic salmon reared under SimFarm and SimNat conditions or captured from the wild.** (A) Compactum thickness (normalized to the spongiosum area), (B) lumen volume as a percentage of ventricular volume and (C) lumen surface area (normalized to lumen volume) (individual data points and means±s.e.m.). LME model statistics are given in each panel and lowercase letters represent Tukey *post hoc* differences between groups.

Tissue composition and lumen properties of hearts from wild, SimNat and SimFarm smolt were assessed by MRI. The compactum thickness (normalized to the spongiosum area) was greatest in SimFarm fish compared with both SimNat and wild fish (*P*<0.001, for both), indicating growth of the compactum in SimFarm fish. Meanwhile, relative compactum thickness was not different between wild and SimNat fish (*P*=0.37, Fig. 3A).

Wild fish had the highest percentage of lumen volume (normalized to ventricular volume) and the lowest lumen surface (normalized to lumen volume) compared with both experimental groups (SimNat: *P*=0.001 and *P*=0.01, respectively and SimFarm: *P*=0.01 and *P*=0.001, respectively). There were no significant differences between SimNat and SimFarm groups in either volume (*P*=0.4) or lumen surface (*P*=0.53; Fig. 3B,C).

### Cardiac function

Systolic and diastolic cardiac function were assessed in 10 SimNat and 10 SimFarm individuals with echocardiography (Fig. S2). The heart rates for SimFarm and SimNat were 75.9±3.8 and 75.8±6.9 beats min^−1^, respectively.

#### Diastolic function

**Fig. 4.**
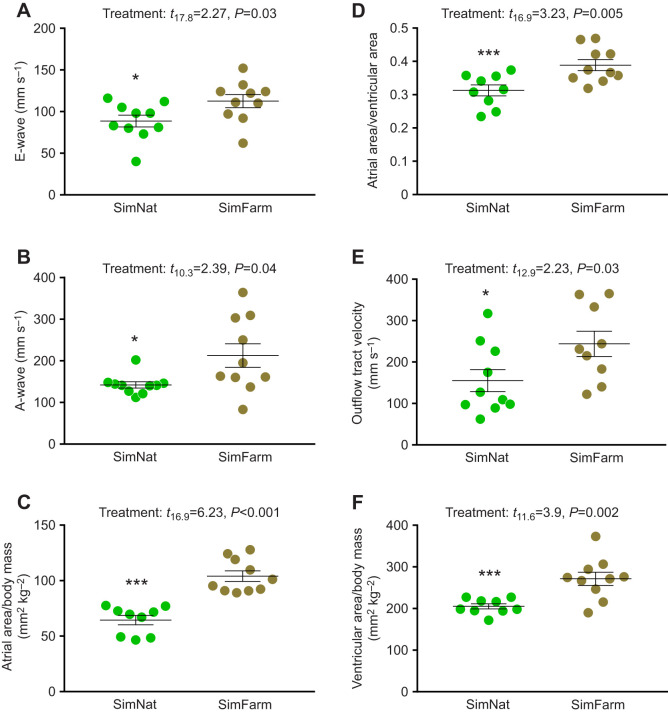
**Echocardiography measurements taken at the end of the smoltification period in Atlantic salmon reared under SimFarm and SimNat conditions.** (A) The early diastolic atrio-ventricular valve wave (E-wave) velocity, (B) the late diastolic atrio-ventricular valve wave (A-wave) velocity, (C) the relative atrial area to body mass ratio, (D) the atrial area relative to the ventricular area, (E) the outflow tract velocity and (F) the relative ventricular area to body mass. Statistics are given in each panel and significant differences between groups are indicated by asterisks (*P<0.05, ***P<0.0001).

Both in the early, E-wave (*t*_17.8_=2.27, *P*=0.03; Fig. 4A) and late, A-wave (*t*_10.3_=2.39, *P*=0.04, Fig. 4B), diastolic blood flow velocities were higher in SimFarm than in SimNat fish. As both E- and A-wave flow velocities were elevated in SimFarm fish, the E:A ratios were not different between SimFarm and SimNat fish (*t*_12.8_=−0.05, *P*=0.96;  Fig. S5A). Isovolumetric relaxation time and the relaxation time per heart beat were also similar between groups (*t*_13.3_=0.69, *P*=0.5 and *t*_13.6_=0.56, *P*=0.6, respectively; Fig. S5B,C), although relative atrial area was increased (both relative to body mass: *t*_11.6_=3.9, *P*=0.002 and to the ventricular area: *t*_16.9_=3.23, *P*=0.005) in SimFarm fish (Fig. 4C,D).

#### Systolic function

There were no significant differences (*t*_17.7_=−0.64, *P*=0.5) in the outflow tract diameter (Fig. S5D) or the pressure gradients across the VB valve (*t*_9.78_=1.68, *P*=0.12; Fig. S5E). Maximal outflow tract blood velocities (*t*_12.9_=2.23, *P*=0.03) were higher in SimFarm individuals (Fig. 4E) whereas the blood velocity time integral was not significantly different between groups (*t*_16.7_=1.39, *P*=0.18; Fig. S5F). Consequently, cardiac output (*t*_15.9_=1.85, *P*=0.08) and stroke volume (*t*_15.8_=1.84, *P*=0.08) were similar between the groups (Fig. S5G,H). Consistent with the higher CSI, ventricular area was observed to be larger (*t*_11.6_=3.9, *P*=0.002) in SimFarm fish (Fig. 4F).

#### Morphology–function relationship

**Fig. 5.**
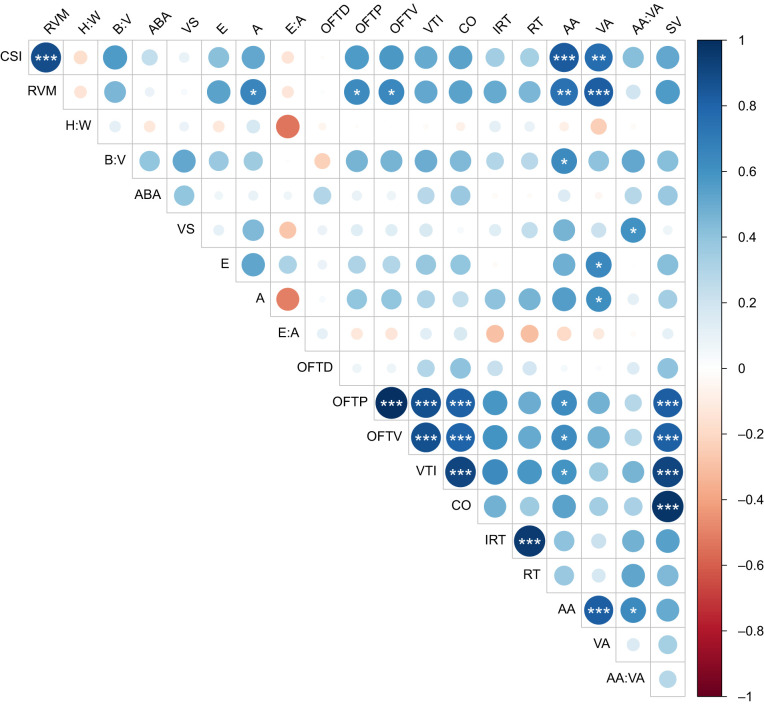
**Correlation analysis of the relationships between cardiac morphology and function.** Spearman's correlation matrix with Benjamini–Hochberg *P*-value correction for multiple comparisons between the cardiosomatic index (CSI), the relative ventricle mass (RVM), the ventricular height:width ratio (H:W), the bulbus:ventricular ratio (B:V), the alignment of the bulbus arteriosus (ABA), the ventricular symmetry (VS), the early diastolic atrio-ventricular valve velocity (E), the late diastolic atrio-ventricular valve wave velocity (A), the ratio between E and A (E:A), the outflow tract diameter (OFTD), the outflow tract pressure (OFTP), the outflow tract velocity (OFTV), the blood velocity time integral (VTI), the cardiac output (CO), the isovolumetric relaxation time (IRT), the relaxation time (RT), the atrial area (AA), the ventricular area (VA), the AA:VA ratio and the stroke volume (SV). Positive and negative correlations are illustrated with a blue and red gradient, respectively, and the magnitude of the correlation is indicated by the size of the circles within each square. Adjusted significant correlations are indicated by asterisks (*P<0.05, **P<0.001, ***P<0.0001).

MRI recordings showed that the increased CSI appears to be caused at least partly by hypertrophied ventricular compact tissue in SimFarm fish. To reveal relationships between cardiac morphology and function, correlation analyses were performed (Fig. 5). First, we observed that CSI correlated with the relative atrial (ρ=0.74, *P*=0.003) and ventricular (ρ=0.82, *P*<0.001) areas. Furthermore, the RVM correlated positively with outflow tract pressure gradient (ρ=0.64, *P*=0.02) and velocity (ρ=0.65, *P*=0.02). Additionally, a positive correlation between RVM and A-wave velocity (ρ=0.65, *P*=0.02) was observed. While the ventricular area correlated with the E-wave velocity (ρ=0.65, *P*=0.02) and A-wave velocity (ρ=0.61, *P*=0.03), atrial area correlated positively with relative bulbus width (ρ=0.64, *P*=0.03), outflow tract pressure gradient (ρ=0.63, *P*=0.03) and velocity (ρ=0.63, *P*=0.03), as well as the velocity time integral (ρ=0.6, *P*=0.04). Taken together, these results indicate that larger (hypertrophied) ventricles facilitate accelerated blood flow velocities in diastole and systole alike. Somewhat surprisingly, enlarged atria seem to only be associated with faster systolic blood flow velocities, stroke volume and ventricular asymmetry (ρ=0.64, *P*=0.02).

## DISCUSSION

In this study, we demonstrate the pivotal role of environmental signals (i.e. photoperiod and temperature) in shaping heart morphology during early rearing stages of Atlantic salmon. Specifically, our findings highlight that rearing conditions mimicking a natural environment (SimNat), characterized by colder water temperatures and a more natural light regime, facilitate the development of an elongated, pyramid-like heart shape with a symmetrical structure, in comparison with SimFarm conditions. In contrast, SimFarm rearing conditions are associated with the development of a heart with a rounded, asymmetrical morphology that deviates significantly from that of the wild salmon heart. This distinct cardiac morphology has previously been linked to impaired cardiac and physical performance in rainbow trout (Claireaux et al., 2005), which is in agreement with our results. Specifically, we found that the morphology observed in SimFarm fish is associated with increased blood flow velocity in the bulbus, which implies a higher cardiac workload. Our results underscore the significance of environmental factors during early life stages as fundamental drivers of this geometrically advantageous heart shape. Further, we observed that specific morphological differences between our treatment groups emerged early on and persisted throughout the experiment. Conversely, other variations appeared later, suggesting that different environmental factors may be time specific in their impact on cardiac morphological traits.

At smoltification, heart morphology differed dramatically between SimNat and SimFarm salmon, with SimNat hearts being more similar to those of wild salmon smolt. For example, the ventricles of SimNat fish were more elongated (higher ventricular height:width ratio) than those of SimFarm fish but more rounded compared with wild smolt. These results agree with previous observational studies showing that sea farmed Atlantic salmon and rainbow trout have rounded ventricles compared with wild specimens (Engdal et al., 2024; Poppe et al., 2003). Moreover, in agreement with our data, rounded ventricles have previously been associated with elevated rearing temperatures during early life stages in commercial hatcheries (Brijs et al., 2020; Frisk et al., 2020). Notably, unlike their wild counterparts, farmed salmon appear to develop increasingly rounded ventricles as they progress through their life cycle. Specifically, we found that mean height:width ratios of SimNat fish were consistently lower than those observed in wild salmon. Nonetheless, these ratios remained stable throughout the freshwater stage, while those of SimFarm fish exhibited a different trend. That is, at the fry stage, SimFarm fish already had lower height:width ratios (approximately 0.9) than SimNat and wild fish, which decreased even further (∼0.6–0.7) at the parr and smolt stage. These differences and changes in ventricle roundness indicate not only that environmental conditions have dramatic effects on ventricular shape but also that environmental conditions in the earliest rearing stages (i.e. before fry stage) are important. In the aquaculture industry, it is common to use rearing temperatures far exceeding those experienced by developing embryos in nature (Handeland et al., 2003) and future research should examine the impact of rearing temperature during embryogenesis on ventricular shape and function. Importantly, cardiac roundness has previously been associated with impaired cardiac and physical performance in rainbow trout (Claireaux et al., 2005) as well as increased mortality risk in Atlantic salmon (Frisk et al., 2020).

Possible mechanisms underlying the observed variation in tissue composition and heart shape remain uncertain. Several internal and external factors are known to induce cardiac remodeling and directly influence heart shape both in fish and in mammals. For example, an increase in the thickness of the compact myocardium may represent an adaptive response to environmental changes in temperature (Graham and Farrell, 1992; Keen et al., 2016) or a pathological reaction to cardiac overload (Johansen et al., 2011, 2017). In this context, increased compactum thickness in SimFarm hearts could reflect remodeling in response to consistently elevated temperatures. This would be in agreement with studies on trout which show that warm acclimation is associated with an increased proportion of the compact myocardium (Farrell et al., 1988), which is believed to compensate for the loss in function associated with elevated temperatures (Farrell et al., 1996).

Alternatively, tissue remodeling in SimFarm fish could reflect increased cardiac workload. Indeed, when the heart faces increased workload, due to either physiological or pathological factors, it responds by hypertrophic growth of the compact myocardium (Graham and Farrell, 1992; Johansen et al., 2011, 2017; Keen et al., 2016). There are several factors that could increase the workload of the heart, including changes in blood flow and viscosity as well as chronically elevated levels of stress hormones. In the present study, differences in tissue remodeling were probably not caused by differences in blood viscosity as a result of increased red blood cell numbers. On the contrary, Hct was not different between groups. Colder temperatures also increase blood viscosity and cardiac workload, but this has been previously associated in rainbow trout with growth of the spongious tissue (Klaiman et al., 2011). Notably, the same remodeling has not been observed in Atlantic salmon (Porter et al., 2022).

Hypertrophic growth of the compact myocardium and remodeling of the ventricular shape have also been shown to be triggered by endocrine factors such as cortisol (Johansen et al., 2011). Indeed, cortisol plays an important role during smoltification and a peak in this hormone during the parr–smolt transformation has been linked to the remodeling of several organs (McCormick, 2012). Previous research has also indicated that cortisol induces hypertrophic growth of the compactum in rainbow trout (Johansen et al., 2017). In the present study, cortisol levels in general did not differ between the experimental groups, except for one time point. That is, SimFarm fish had lower cortisol levels than SimNat fish at the start of the smoltification period. Thus, differences in cortisol levels between treatment groups do not explain the observed differences in heart morphologies throughout the freshwater period.

Lastly, the deviating heart shape of SimFarm fish could be expected to affect the workload of the heart and in that way induce tissue remodeling. In particular, we found that SimFarm fish exhibited considerably enlarged bulbi in comparison with both SimNat and wild fish from parr to the end of the smoltification period. This enlargement could imply alterations in the elastic properties of the bulbus arteriosus, which is part of the outflow tract of the teleost heart and extends from the ventricle to the ventral aorta. The bulbus serves to steady pulsatile blood flow across the capillary networks of the gill and protects the gill capillaries from high systolic pressure (Evans et al., 2013). In other words, if the enlarged bulbus is also less compliant, this may create an obstruction leading to increased blood flow velocity. Notably, using echocardiography, we found that the morphology observed in SimFarm fish is indeed associated with increased blood flow velocity during systole. In addition, both E- and A-wave velocities were elevated during ventricular filling in this group. Of note, geometrical development of the zebrafish heart is known to be influenced by cardiac blood flow (Bartman and Hove, 2005). Thus, accelerated blood flow through the SimFarm heart could in fact contribute to the observed remodeling and altered heart shape. Alternatively, it is likely that an enlarged and remodeled heart produces faster blood flow. Typically, faster blood flow during both diastole and systole is associated with concentric hypertrophy in situations where the ventricular outflow tract is partially obstructed (Agatston et al., 1989; Kenny et al., 1991). In this context, significant correlations between relative ventricle mass and area with both diastolic and systolic blood flow velocities indicates that the SimFarm fish heart is producing faster blood flow.

We also observed that SimFarm fish develop more misaligned bulbi compared with wild fish, in agreement with previous literature (Engdal et al., 2024; Poppe et al., 2003). Interestingly, Brjis et al. (2020) observed that the ability of rainbow trout to tolerate confinement stress was correlated with a lower bulbus arteriosus angle alignment, which suggests that this angle may play a role in cardiac stress resilience. A misaligned and less compliant bulbus is thus expected to increase the load on the heart.

In summary, we suspect that the morphology observed in SimFarm fish is related to higher cardiac workload as implicated by increased blood flow velocities. We can, however, not exclude the possibility that other environmental or endocrine factors have contributed to the observed differences between the experimental groups. Therefore, future studies should address physiological and molecular mechanisms underlying geometrical remodeling of the salmon heart. Also, whereas no differences in fish performance (growth, mortality, etc.) were observed at these early life stages, it is tempting to speculate that the morphological and functional differences observed may lead to cardiac disorders later in life, which should also be addressed in future studies.

To the best of our knowledge, lumen volume and surface area data have not been previously reported for any teleost fish species. In the current study, we employed high-resolution MRI scans to quantitatively assess these parameters in both experimental and wild salmon. While lumen volume and surface area remained comparable between the two experimental groups (SimFarm and SimNat), the substantial disparities observed between wild and experimental fish warrant further consideration. Notably, the ventricular lumen volume in experimental smolt (comprising both SimFarm and SimNat groups) was less than half that of their wild counterparts. This drastic reduction in lumen volume appears to be directly associated with a substantial, albeit not proportionate, increase in lumen surface area in the same fish. Given that measurements of these lumen characteristics have not been previously documented, we are limited to speculation regarding their underlying causes and significance. However, we speculate that an augmentation in lumen surface area may potentially arise from increased branching of the spongy myocardium. This tissue relies solely on venous blood returning to the heart for oxygenation and the increased surface area may signify an elevated demand for oxygen or nutrients. This, however, probably contributes to a reduction in lumen volume and possibly enhances blood turbulence within the heart. It is difficult to imagine how this remodeling would be inherently adaptive, except through providing increased surface area for enhanced resource uptake. If this holds true, it implies that experimental fish experienced specific stimuli in their rearing conditions, necessitating such compensatory remodeling. While this finding is undoubtedly intriguing and merits careful consideration, it is beyond the scope of this article to speculate on the collective influences of various rearing conditions on the observed differences in internal cardiac characteristics.

### Methodological considerations of the study

Despite our efforts to mimic natural conditions for the SimNat fish, rearing conditions for wild and experimental fish differed considerably. Experimental fish were confined indoors in controlled tank environments, with reduced possibility for physical activity and unlimited access to food, in stark contrast to the conditions of their wild counterparts. This was also reflected in morphological differences between wild and SimNat fish. These differences may have been associated with other factors affecting cardiac remodeling. For example, the elongated pyramid-shaped ventricle appears to be adapted for physical activity, and thus increased training may play a crucial role in cardiac shape. However, further investigations are needed to explore this aspect.

Of note, SimNat fish were transferred from 7 to 10°C to be able to assess cardiac function at the same temperature across groups. Thus, only SimNat fish, and not SimFarm fish, experienced a temperature change prior to functional testing. Although we believe that 2 days of acclimation to experimental temperature should be sufficient to avoid acute effects of temperature change, we cannot exclude that this difference may have affected heart function. In addition, MS-222 was used as an anesthetic during functional measurements, which has been shown to reduce cholinergic inhibition, thereby causing elevated heart rate. However, as the two groups were examined with echocardiography under the same experimental conditions, we believe that our results reflect a true difference in the functional characteristics between SimNat and SimFarm fish and that these differences can probably be extrapolated to free-swimming individuals.

### Conclusions

SimFarm fish are characterized by a rounded ventricle, thicker compactum and increased misalignment of the bulbus arteriosus, compared with both SimNat and wild fish. These morphological differences are associated with increased diastolic and systolic blood flow velocities in SimFarm fish. Together with the MRI results, this is indicative of hemodynamic alterations due to cardiac hypertrophy, consistent with those typically observed in hypertrophied mammalian hearts (Kenny et al., 1991). However, more comprehensive studies of the potential implications of altered cardiac morphology on cardiac function are needed. Importantly, our findings have implications reaching beyond the industrial farming of salmonids. Our results are in agreement with other studies which report that elevated temperatures impact heart morphology in a manner that is associated with impaired cardiac and physical performance (Brijs et al., 2020; Claireaux et al., 2005). These findings highlight the potential risk posed by climate change on the fitness of wild salmon populations. Thus, more research should be conducted to understand how and why the environment promotes such morphological remodeling of the heart.

## Supplementary Material

10.1242/jexbio.247557_sup1 Supplementary information

Dataset 1. Raw data
