# Supplementary material for: Importance of environmental signals for cardiac morphological development in Atlantic salmon
Source: J Exp Biol. 2024 Oct 18;227(20):jeb247557. doi: 10.1242/jeb.247557 (PMC11529873; doi:10.1242/jeb.247557)
Supplement: Supplementary information [file jexbio-227-247557-s1.pdf]

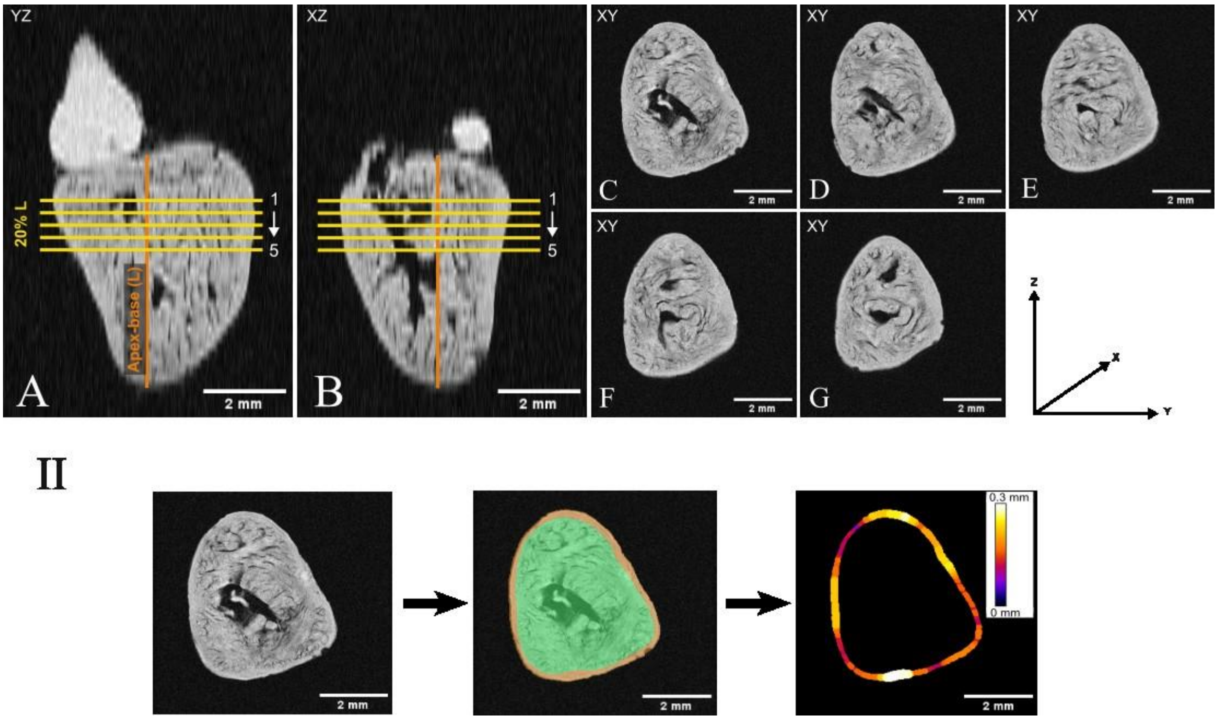

Fig. S1. Compactum thickness analyses. I. MRI scans of the hearts were acquired at XYZ resolution of 25 x 25 x 200 µm. Five XY slices were chosen for the analyses: slice 1 was chosen at the widest part of the ventricle (slice 1 in **A** and **B**; corresponding image in **C**), and slices 2-5 (**A** and **B**) were selected within 20% of the total length of the ventricle (apex-base, L) towards the apex of the ventricle, resulting in additional four slices separated by 5% of the ventricular length (**D**-**E**). Compactum thickness was then analyzed in the five selected slices (**C-E**) and normalized to the square root of the spongious area in each slice as described in the main text. The average of the compactum thickness in the five slices was used for further statistical analyses. **II.** A schematic illustration of the analysis: selection of compactum (orange) and spongiosum (green), and the corresponding local thickness map showing the local thickness of compactum across the ventricular section. The mean compactum thickness was calculated as an average pixel value in the local thickness map.

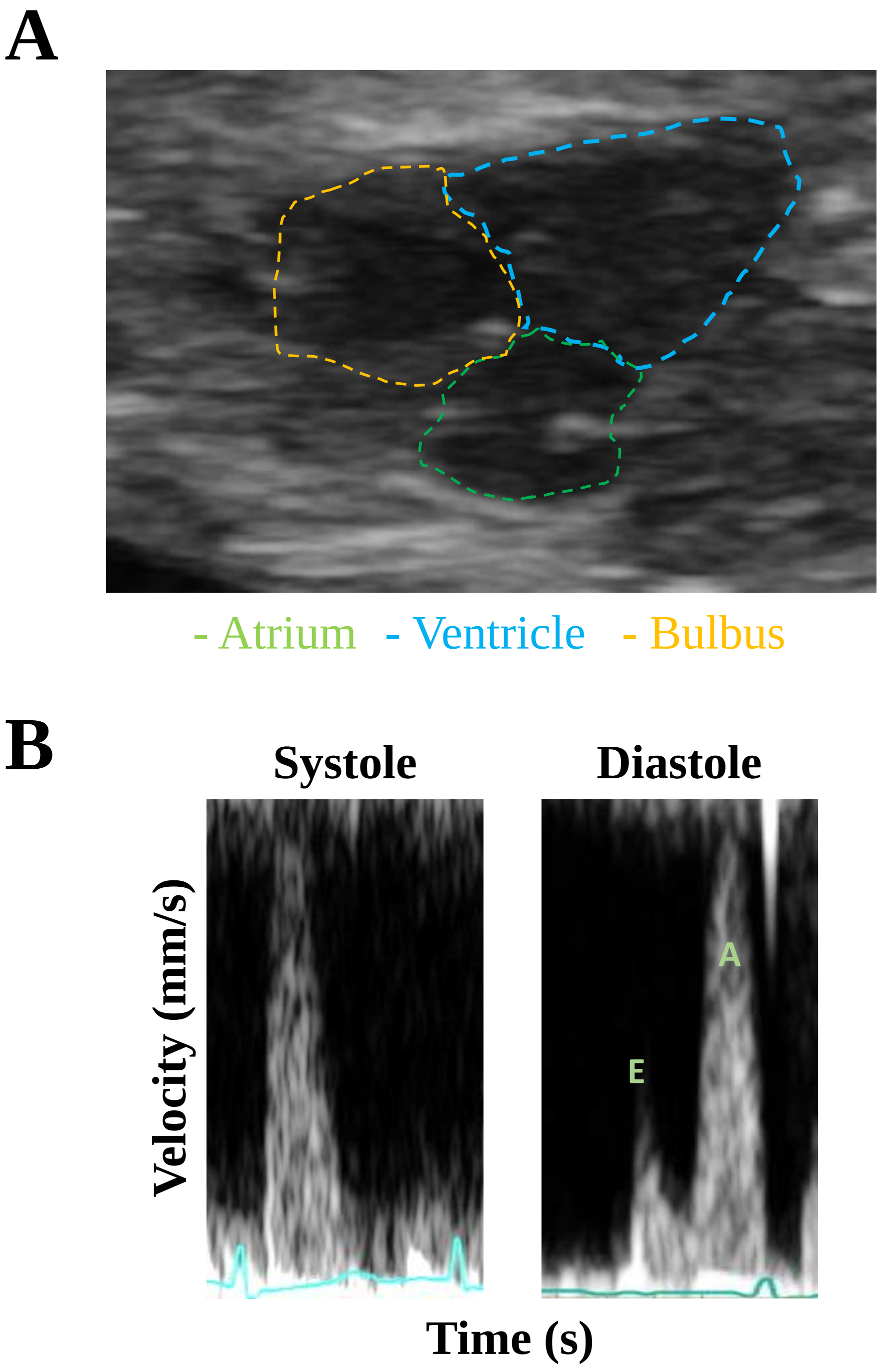

Fig. S2. Echocardiography in salmon smolt. A. Representative 2D ultrasound image of the heart. Atrium, ventricle, and bulbus are indicated with dashed lines. **B.** Using pulsed wave doppler, systolic (left) and diastolic (right) hemodynamics were recorded at the ventrico-bulbar valve and atrio-ventricular valve, respectively. The early (E) and late (A) diastolic waves are indicated in the diastolic trace.

Journal of Experimental Biology • Supplementary information

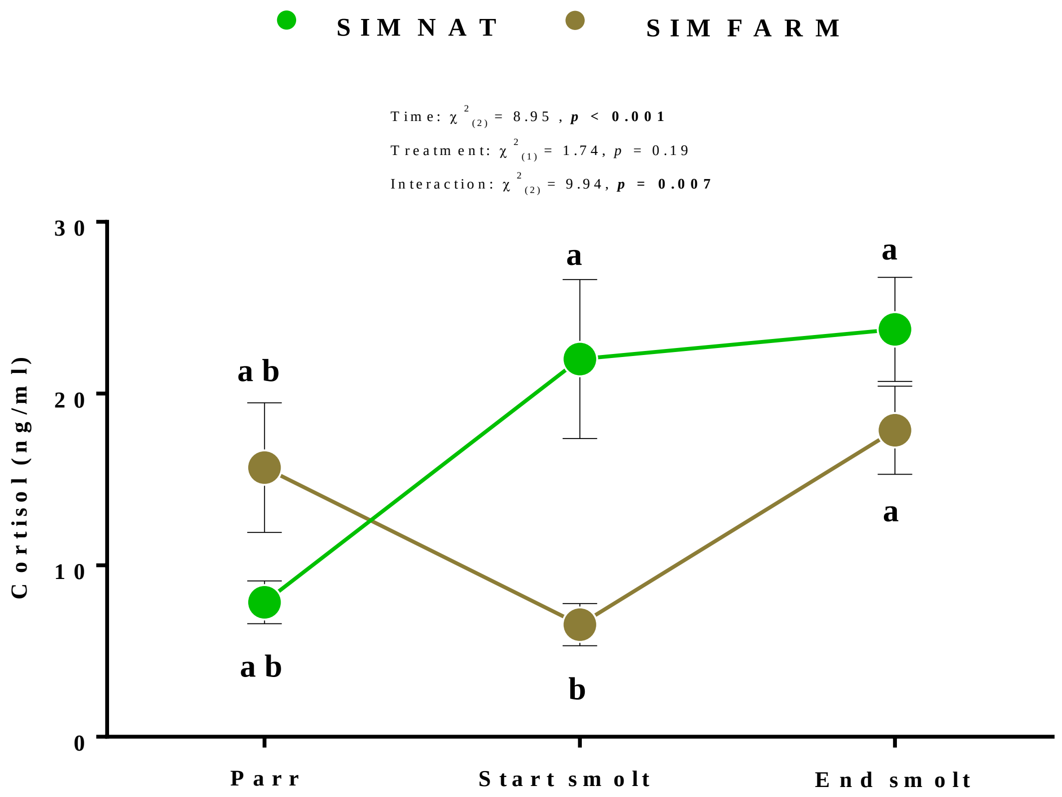

Fig. S3. Mean plasma cortisol (± SEM) levels throughout the freshwater life stages (parr, start and end of smoltification) for Atlantic salmon reared under simulated farmed (SIMFARM) or simulated natural (SIMNAT) conditions. Linear Mixed Effects model statistics are given in each panel and small letters represent Tukey post-hoc differences between groups at all timepoints.

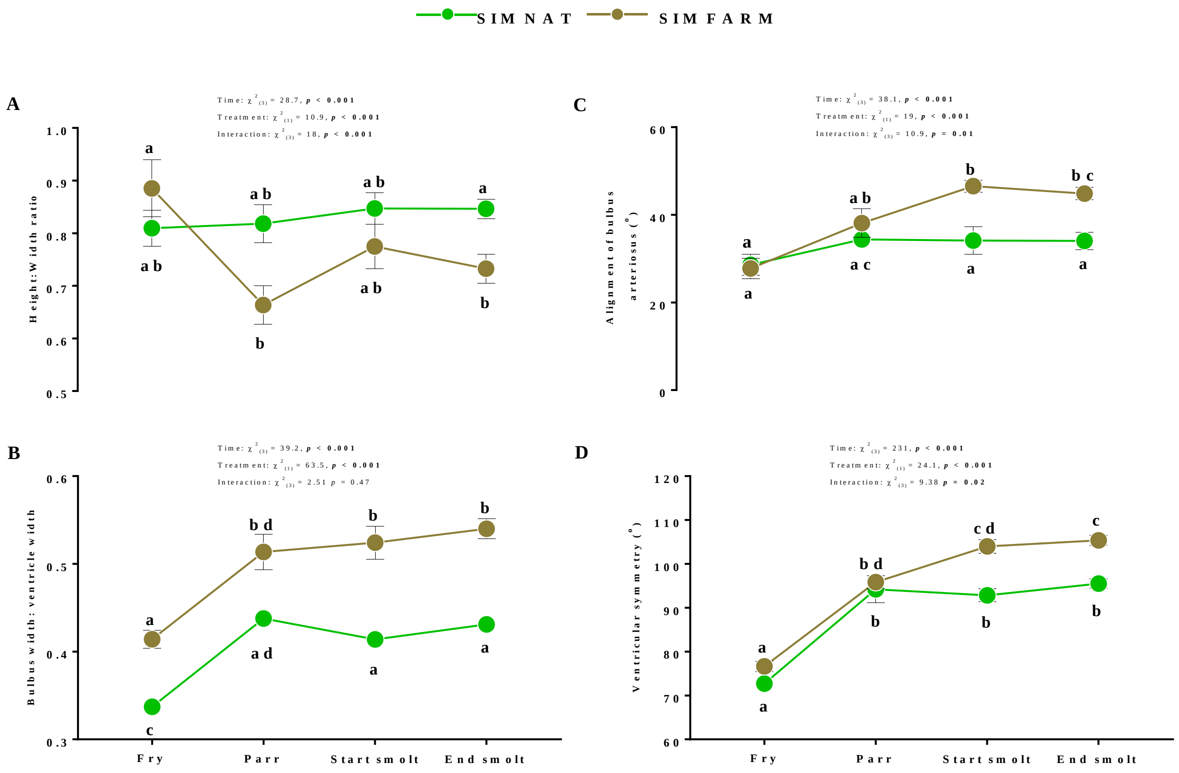

Fig. S4. Heart morphology average (± SEM) measurements throughout the freshwater life stages (fry, parr, start and end of smoltification) for Atlantic salmon reared under simulated farmed (SIMFARM) or simulated natural (SIMNAT) conditions. Linear Mixed Effects model statistics are given in each panel and small letters represent Tukey post-hoc differences between groups at all timepoints.

Journal of Experimental Biology • Supplementary information

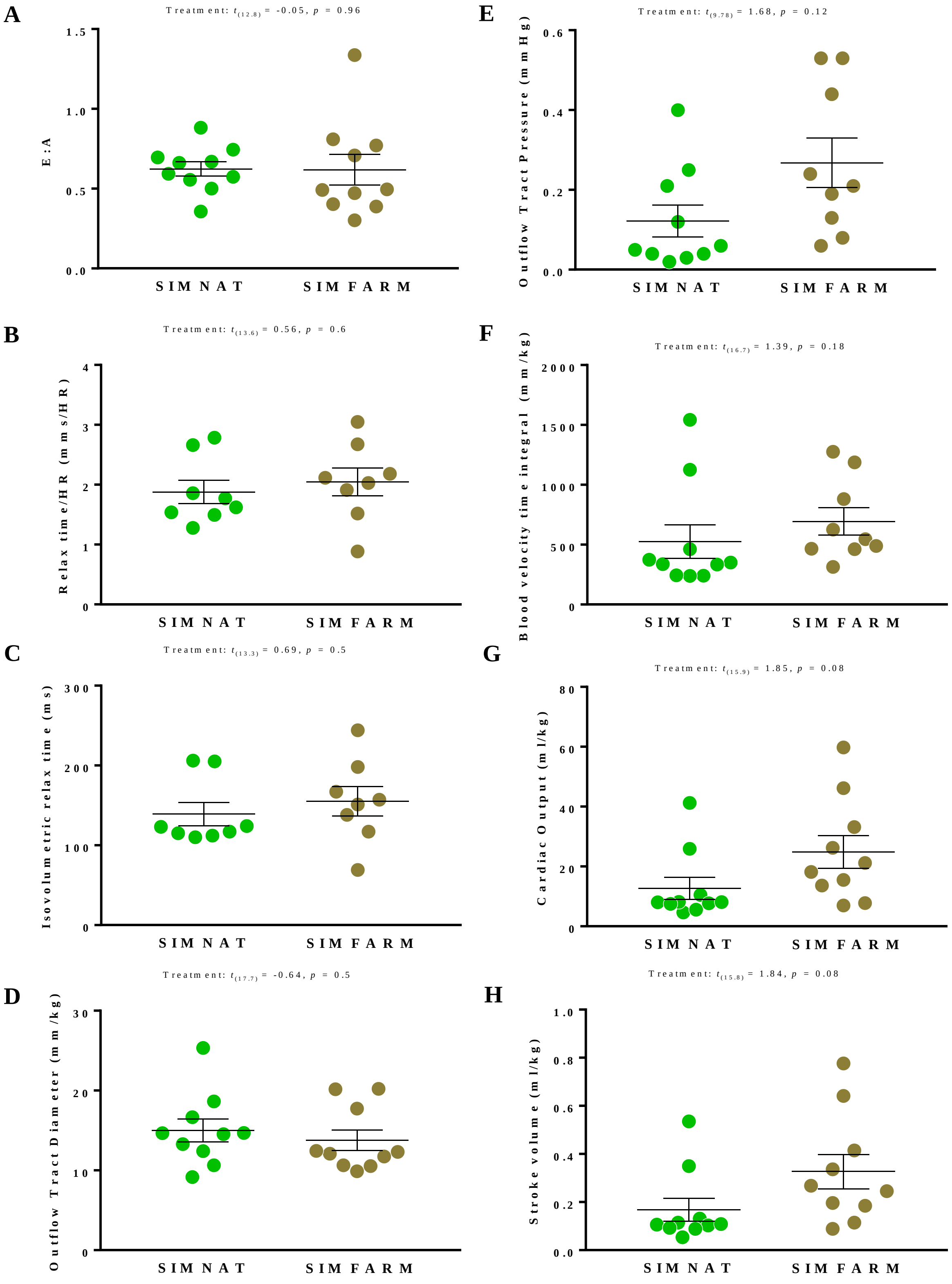
A
Treatment: $t_{(12.8)}$ = -0.05, p = 0.96
E:A
1.5
1.0
0.5
0.0
SIMNAT
SIMFARM
B
Treatment: $t_{(13.6)}$ = 0.56, p = 0.6
Relax time/HR (ms/HR)
4
3
2
1
0
SIMNAT
SIMFARM
C
Treatment: $t_{(13.3)}$ = 0.69, p = 0.5
Isovolumetric relax time (ms)
300
200
100
0
SIMNAT
SIMFARM
D
Treatment: $t_{(17.7)}$ = -0.64, p = 0.5
Outflow Tract Diameter (mm/kg)
30
20
10
0
SIMNAT
SIMFARM
E
Treatment: $t_{(9.78)}$ = 1.68, p = 0.12
Outflow Tract Pressure (mmHg)
0.6
0.4
0.2
0.0
SIMNAT
SIMFARM
F
Treatment: $t_{(16.7)}$ = 1.39, p = 0.18
Blood velocity time integral (mm/kg)
2000
1500
1000
500
0
SIMNAT
SIMFARM
G
Treatment: $t_{(15.9)}$ = 1.85, p = 0.08
Cardiac Output (ml/kg)
80
60
40
20
0
SIMNAT
SIMFARM
H
Treatment: $t_{(15.8)}$ = 1.84, p = 0.08
Stroke volume (ml/kg)
1.0
0.8
0.6
0.4
0.2
0.0
SIMNAT
SIMFARM

Fig. S5. Echocardiography measurements of, the ratio between the early and the late (E:A) diastolic atrio-ventricular valve wave velocity (**A**), the cardiac relax time corrected for heart rate (**B**), the isovolumetric relax time (**C**), the outflow tract diameter (**D**), outflow tract pressure (**E**), the blood velocity time integral (**F**), the cardiac output (**G**) and the stroke volume (**H**), taken at the end of the smoltification period in Atlantic salmon reared under simulated farmed (SIMFARM) or simulated natural (SIMNAT) conditions. Statistics are given in each panel and * represent significant differences between groups.

Dataset 1. Raw data

Available for download at
https://journals.biologists.com/jeb/article-lookup/doi/10.1242/jeb.247557#supplementary-data

Journal of Experimental Biology • Supplementary information
